# A Small Guanosine Triphosphate Binding Protein *PagRabE1b* Promotes Xylem Development in Poplar

**DOI:** 10.3389/fpls.2021.686024

**Published:** 2021-06-04

**Authors:** Ying-Li Liu, Li-Juan Wang, Yu Li, Ying-Hua Guo, Yuan Cao, Shu-Tang Zhao

**Affiliations:** ^1^State Key Laboratory of Tree Genetics and Breeding, Research Institute of Forestry, Chinese Academy of Forestry, Beijing, China; ^2^Co-innovation Center for Sustainable Forestry in Southern China, Nanjing Forestry University, Nanjing, China; ^3^Key Laboratory of Plant Molecular Physiology, Institute of Botany, Chinese Academy of Sciences, Beijing, China

**Keywords:** poplar, small gtp binding protein, *PagRabE1b*, cell wall, wood formation

## Abstract

Rab GTPases are the subfamily of the small guanosine triphosphate (GTP)-binding proteins which participated in the regulation of various biological processes. Recent studies have found that plant Rabs play some specific functions. However, the functions of *Rabs* in xylem development in trees remain unclear. In this study, functional identification of *PagRabE1b* in *Populus* was performed. Quantitative reverse transcription PCR (qRT-PCR) results showed that *PagRabE1b* was highly accumulated in stems, especially in phloem and xylem tissues. Overexpression of *PagRabE1b* in poplar enhanced programmed cell death (PCD) and increased the growth rate and the secondary cell wall (SCW) thickness. Quantitative analysis of monosaccharide content showed that various monosaccharides were significantly increased in secondary xylem tissues of the overexpressed lines. Flow cytometry analysis revealed that the number of apoptotic cells in *PagRabE1b-OE* lines is more than a wild type (WT), which indicated that *PagRabE1b* may play an important role in PCD. Further studies showed that overexpression of *PagRabE1b* increased the expression level of genes involved in SCW biosynthesis, PCD, and autophagy. Collectively, the results suggest that *PagRabE1b* plays a positive role in promoting the xylem development of poplar.

## Introduction

Trees are the most abundant natural sources important for sustainable energy and the sinks of atmospheric carbon dioxide (Zhang et al., 2015; Zhong and Ye, 2015). Wood biomass is mainly composed of the secondary cell wall (SCW) and is widely used in many applications, such as house construction, biofuels, pulping, and paper-making. Because of the substantial economic value of wood, elucidating the molecular regulatory mechanism of wood formation will be useful for the manipulation of wood quality and quantity through molecular breeding.

The development of SCW is a complicated procedure requiring synchronization of several regulatory and metabolic pathways. Previous studies have revealed that the SCW biosynthesis is mainly regulated by transcriptional regulatory networks of NAC and MYB transcription factor (TF) families (Zhong and Ye, 2015). During SCW formation, several NAC TFs are considered as master switches and in the top layer of the SCW regulatory network (Xie et al., 2018). A series of additional TFs, such as *MYB46* and *MBY83*, belong to the second layer of a transcriptional network of the SCW formation. In addition, several other MYB TFs in the third layer have been identified as direct targets of *MYB46/83* (Zhang et al., 2018a); most of these MYB members positively regulate SCW biosynthesis. SCWs mainly consist of cellulose, hemicelluloses, and lignin. Cellulose taking about 40–50% of wood components is glucose polymers. Hemicelluloses constitute a major part of lignocellulosic biomass, mainly including xyloglucans, xylans, mannans, glucomannans, and β-(1→ 3, 1→ 4)-glucans (Scheller and Ulvskov, 2010). Lignin is a complex three-dimensional polyphenolic polymer of *p*-hydroxyphenyl (H), guaiacyl (G), and syringyl (S) lignins (Boerjan et al., 2003). Monolignol biosynthesis is carried out through the common phenylpropanoid pathway, and several central genes involved in lignin synthesis during secondary wall formation in *Populus* had been well-identified (Wang et al., 2018). Finally, the lignified vessel and fiber cells undergo programmed cell death (PCD) for complete lysis of cell content in the maturation of xylem cells (Courtois-Moreau et al., 2009).

Small GTPases also play important roles in the regulation of cell wall development by monitoring cytoskeletal arrangement and membrane trafficking (Oda and Fukuda, 2014). In the eukaryotic, the Ras subfamily of small GTPases comprises five families: Ras, Rab, Ran, Arf, and Rho (Hall, 1998). Rho and Rab of plants play critical roles in the development of primary and SCWs (Oda and Fukuda, 2014). ROPGEF4 and ROPGAP3 mediate Rho GTPase ROP11 to originate the fundamental patterning of SCWs in xylem cells, and then ROP11 interacts withMIDD1 to provoke local depolymerization of cortical microtubules (Oda and Fukuda, 2012). In *Arabidopsis*, a boundary of the ROP domain (BDR1) and Wallin (WAL) complex regulates cell wall development. BDR1 could recruit WAL to the plasma membrane and then regulate an ROP-act in a pathway to shape pit boundaries (Sugiyama et al., 2019). Rab GTPase has also been shown to play important roles in SCW deposition. Overexpression of a constitutively active mutant of *RabG3b* (RabG3bCA) stimulated both autophagy and tracheary element formation in *Arabidopsis* (Kwon et al., 2010) and increased xylem growth due to the stimulation of autophagy during xylem development in *Populus* (Kwon et al., 2011).

The Rab family in plants is categorized into eight subfamilies (RabA-RabH) (Vernoud et al., 2003). We found out that there was a total of 67 *PtRab* genes in *Populus trichocarpa* and were grouped into eight subfamilies (Zhang et al., 2018b). Most of the *PtRab* genes were preferentially expressed in phloem and xylem. During the development of the poplar stem, the majority of *PtRabs* were preferentially expressed in the transition region from primary growth to secondary growth. These results suggested that the *PtRab* genes might participate in the biological processes related to xylem development. In this study, we explored the function of *PagRabE1b* in xylem development using transgenics overexpressing constitutively active mutant and native *PagRabE1b*, which revealed that *PagRabE1b* influenced both SCW biosynthesis and final PCD during wood formation.

## Materials and Methods

### Plant Materials and Growth Conditions

Hybrid poplar (*Populus alba* × *Populus glandulosa, Pag*) clone 84 K was used for gene transformation. Plants were cultivated for 4 weeks under *in vitro* conditions and then transplanted to the soil and grown for 12 weeks in a greenhouse (16-h light: 8-h dark, 25°C: 20°C, with relative humidity in the range 50–60%).

### Vector Construction and Plant Transformation

*PagRabE1b* and *PagRabE1b* (Q74L) (point mutation of Q74 to L in motif 2, a constitutively active form of *PagRabE1b*) genes were cloned into the pMDC32 plant expression vector and transformed into poplars previously (Zhang et al., 2018b). Two overexpression lines of *PagRabE1b* and *PagRabE1b* (Q74L) with high expression levels were selected and named as OE-1 and OE-9 for *PagRabE1b* and QL-8 and QL-13 for *PtRabE1b* (Q74L), respectively.

### Histological Analyses

The basal stems of wild type (WT), OE-1, OE-9, QL-8, and QL-13 plants were sectioned for histology using a vibratome (VT1000S; Leica, Wetzlar, Germany) to a thickness of 50 μm. Then, the sections were stained with 0.05% toluidine blue (TBO) for 60 s at room temperature, rinsed three times in water, and photographed with an Olympus BX51 microscope (Du et al., 2020).

### Wall Thickness Measurement

Secondary cell wall thickness was observed by using confocal laser scanning microscopy (CLSM) and transmission electron microscopy (TEM). The 15th internodes of 2-month-old WT and *PagRabE1b* transgenic plants were sectioned using a vibratome at a thickness of 50 μm. CLSM pictures were captured with an LSM880 microscopy (Zeiss) using 488-nm laser excitation (5% power). TEM observation was implemented as described by Zhao et al. (2020). The 20th internodes from 3-month-old WT and transgenic plants were cut into 2-mm pieces, which were fixed using 2.5% paraformaldehyde and 0.5% glutaraldehyde in PBS (0.1 M, pH 7.4) with vacuum infiltration. The pieces were then washed three times with 0.1 M PBS and further fixed in 1% osmic acid for 2 h at room temperature. Ultrathin sections were made and photographed using an HT-7700 microscope (Hitachi) and a Gatan *ORIUS*^TM^ *SC*1000CCD Camera (Gatan Inc., USA). The cell wall thickness of at least 100 cells from three individual plants of each line was measured using ImageJ software and analyzed statistically using Student's *t*-test.

### Analysis of SCW Composition

The 10th to 20th internodes of 3-month-old WT and *PagRabE1b* transgenic plants were collected and freeze-dried at −60°C on a vacuum freeze. The dried stems were powdered by ball milling and used to measure alcohol-insoluble residues according to Zhao et al. (2020). The monosaccharide content was analyzed through GC-MS (Agilent, Santa Clara, CA, USA). Three biological replicates were analyzed for each line.

### Programmed Cell Death Analysis of Stem-Differentiating Xylem Protoplasts

The *Populus* protoplasts from SDX isolation were carried out as previously described by Lin et al. (2013)and Wang et al. (2020) with minor modifications. Briefly, the debarked stem segments of 3-month-old WT and *PagRabE1b* transgenic poplars were incubated in a cell wall digestion enzyme solution [1.5% (wt/vol) Cellulase R-10 and 0.4% (wt/vol) pectolyase Y-23 in 20-mM MES,0.6-M mannitol and 20-mM KCl solution, 10-mM CaCl_2_, and 0.1% (wt/vol) BSA, pH 5.7] for 40 min in the dark at room temperature. Protoplasts were filtered through a 70-μm cell strainer and spun down at 150 × g for 5 min. Protoplasts were resuspended in W5 solution (2-mM MES, pH 5.7, 125-mM CaCl_2_, 154-mM NaCl, 0.1-M glucose, and 5-mM KCl). The isolated protoplasts were resuspended in a 195-μl prediluted binding buffer, and 5-μl Annexin V-FITC solution (Solarbio, Beijing, China) was added, mixed, and incubated for 30 min at room temperature, and then added 1 μl of the 20-μg ml^−1^ propidium iodide (PI) storage solution. After the addition of another 300-μl binding buffer, the suspended protoplasts were analyzed using a BD Aria SORP cell sorter (BD Biosciences, USA) with 488-nm excitation for FITC and 530 nm for PI. Three independent sets of experiments were performed.

### Gene Expression Analysis

The apex, stems, and roots were harvested from 8-week-old poplars and were sampled for quantitative reverse transcription PCR (qRT-PCR). For sampling, the xylem and phloem–cambium tissues from stems of 8-week-old poplar were debarked and scraped with a sterile double-edged razor blade from the outer of the debarked stem or the inner surface of the peeled bark. To examine the expression levels of marker genes involved in xylem development by qRT-PCR, 2-month-old WT and *PagRabE1b* transgenic plants were debarked, and developing xylems were scraped. Total RNA was extracted from all the samples using the RNeasy Plant Mini Kit (Qiagen, Hilden, Germany) and genomic DNA was eliminated with the on-column treatment of RNase-free DNase I (Qiagen, Hilden, Germany). First-strand complementary DNA (cDNA) was synthesized with about 1-μg RNA using the SuperScript III reverse transcription kit (Invitrogen) according to the instruction of the manufacturer. Primers with annealing temperatures of 58–60°C and amplification lengths of 100–250 bp were designed using Primer3 software (http://primer3.ut.ee/). All primers were listed in Supplementary Table 1. qRT-PCR was carried out using KAPA SYBR FAST qPCR master mixture on Roche LightCycler 480 (Roche Applied Science, Penzberg, Upper Bavaria, Germany) according to the instructions of the manufacturer. All experiments were performed in three biological replicates and three technical replicates. The *PagActin* gene (Potri.001G309500) was used as the internal control (Wang et al., 2016; Zhao et al., 2020).

## Results

### Expression Pattern of *PagRabE1b*

**Figure 1:**
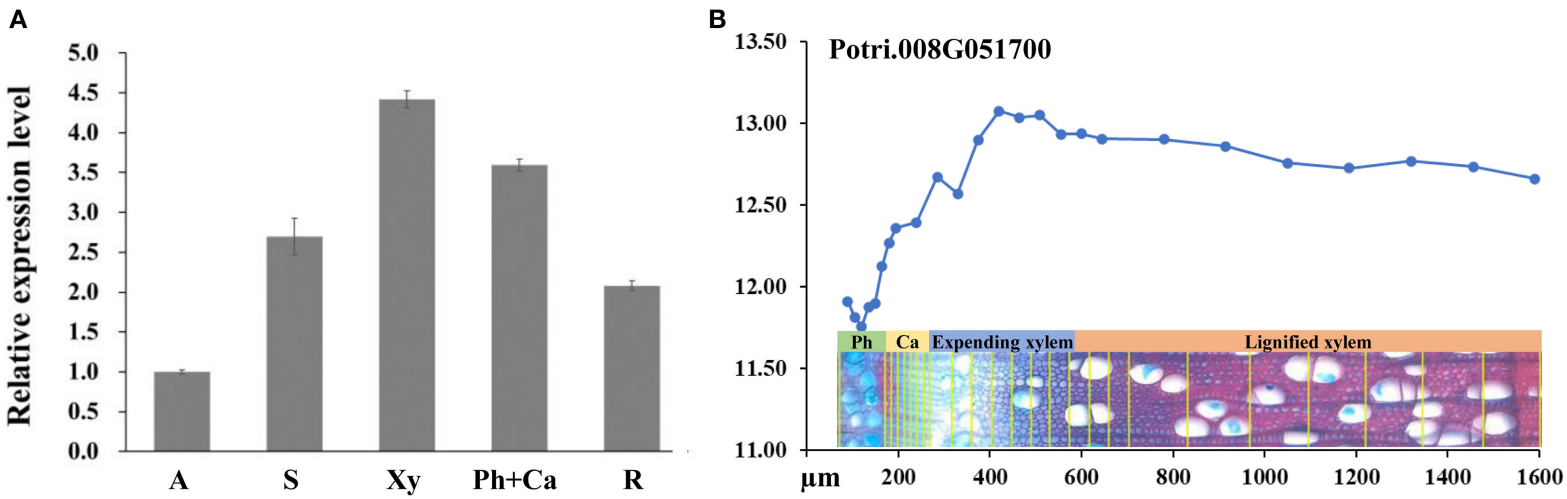
Expression patterns of *PagRabE1b* in poplar. **(A)** Quantitative reverse transcription PCR (qRT-PCR) was carried out to analyze the gene expression levels of *PagRabE1b* indifferent tissues of poplar. The *actin* gene was used as an internal control. **(B)** Expression profiles of *PagRabE1b* among developing phloem, cambium, and developing xylem. Data were derived from the AspWood database (http://aspwood.popgenie.org) (Sundell et al., 2017). A, apex; S, stem; Xy, xylem; Ph, phloem; Ca, cambium; R, root.

To investigate the expression pattern of *PagRabE1b*, qRT-PCR was used to analyze the gene expression in tissues collected from the apex, stems, and roots. The results revealed that *PagRabE1b* expression could be detected in all the tissues and was high in xylem and phloem–cambium (Figure 1A). A similar expression pattern was also observed by analysis of the AspWood database (http://aspwood.popgenie.org) (Sundell et al., 2017), which showed that *PagRabE1b* is highly expressed in expanding xylem (Figure 1B). These results suggested a potential role of *PagRabE1b* in xylem development in poplar.

### Overexpression of *PagRabE1b* Promotes Xylem Development in Transgenic Poplar

**Figure 2:**
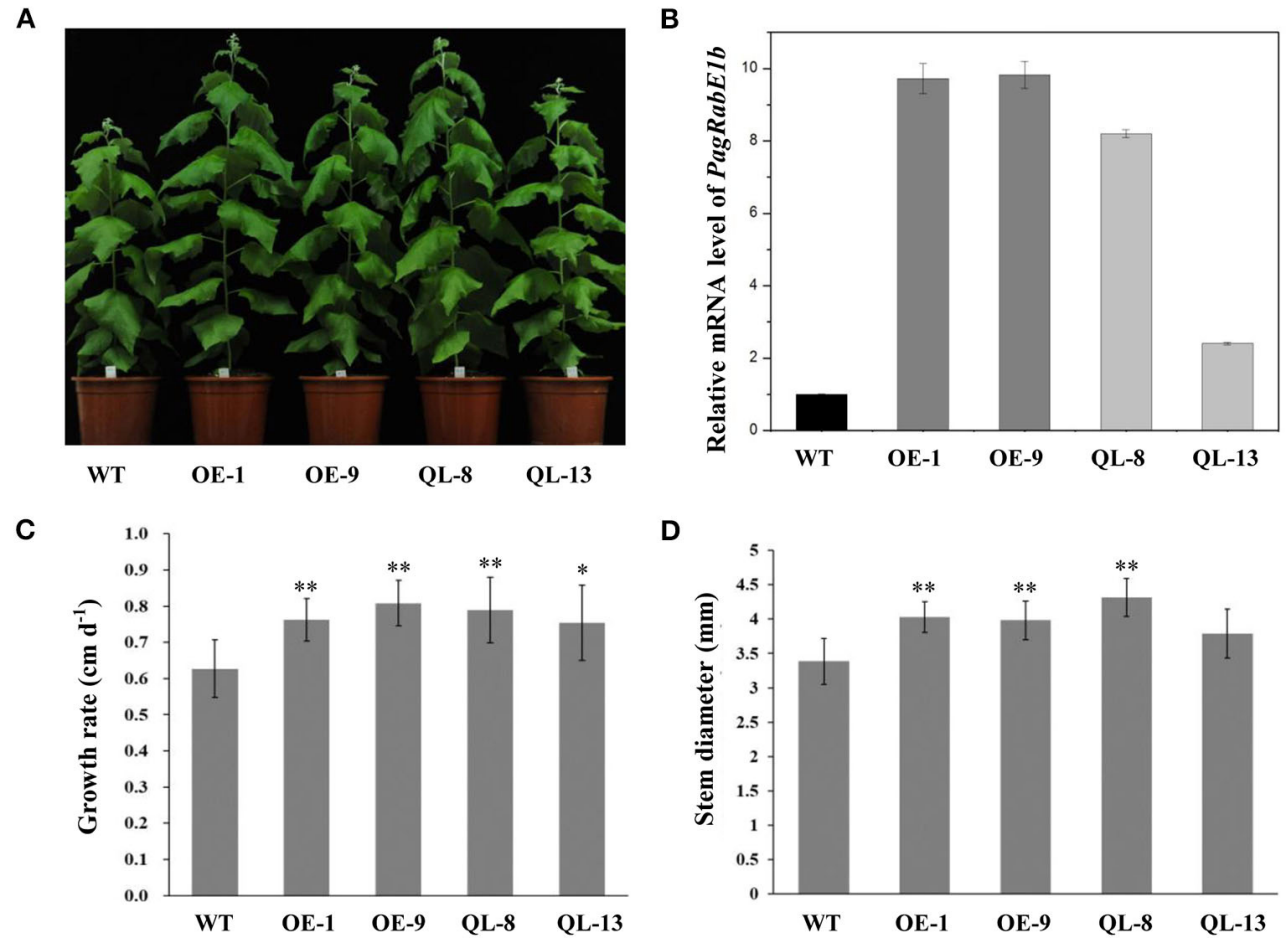
Growth phenotypes of *PagRabE1b* transgenic poplars compared with wild type (WT). **(A)** A photograph of WT and *PagRabE1b* overexpression lines. Bar = 10 cm; **(B)** qRT-PCR analyses of the transcript levels of *PagRabE1b* in WT, OE-1, OE-9, QL-8, and QL-13 lines. **(C,D)** The growth rate and basal diameter of 3-month-old WT, OE-1, OE-9, QL-8, and QL-13 lines. The growth rate and diameter: means ± SD of six clonally propagated plants. Student's *t*-test; *P < 0.05; **P < 0.01.

**Figure 3:**
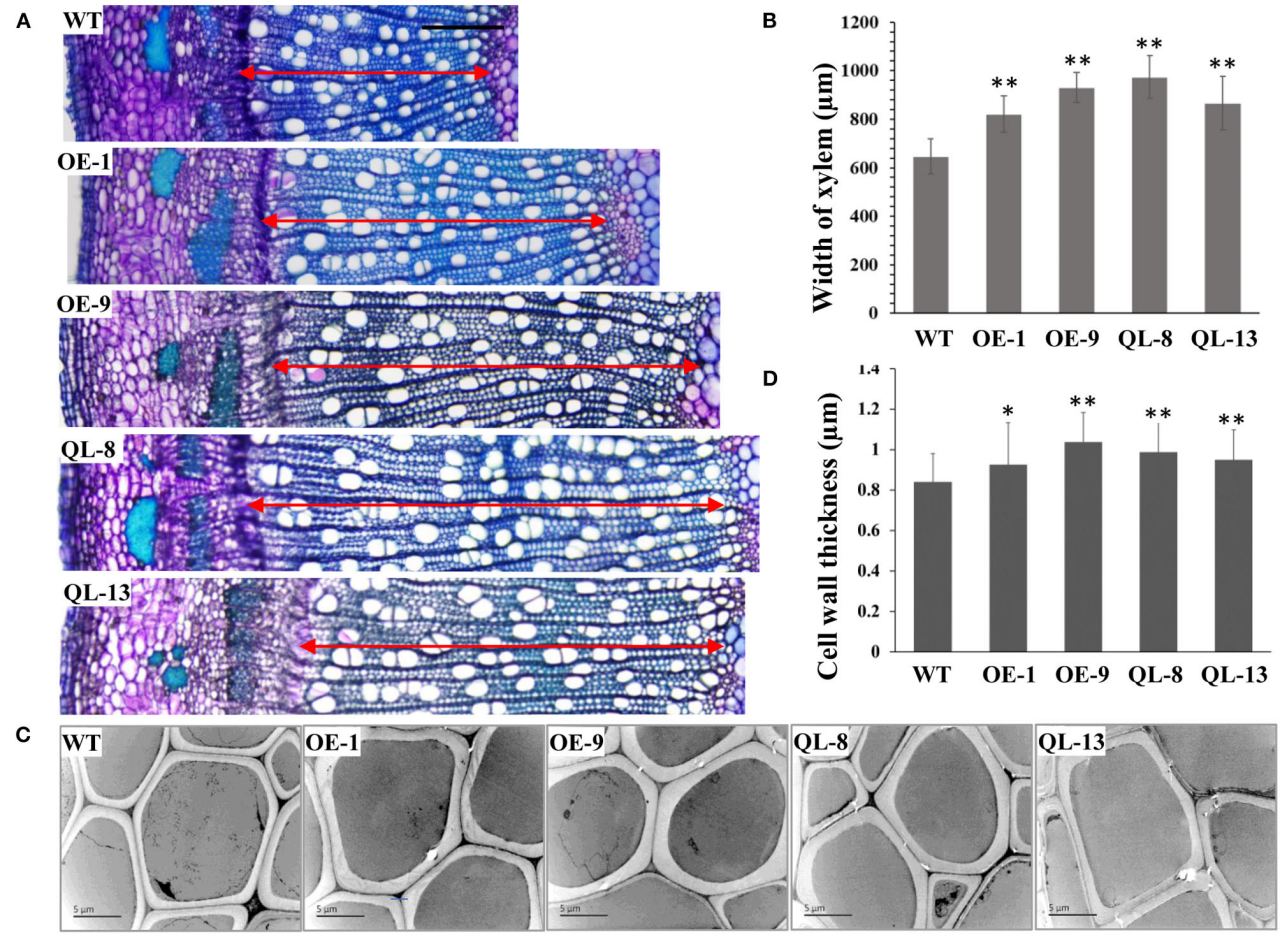
Xylem development was increased by *PagRabE1b* overexpression. **(A)** Transverse sections of the basal stems of 3-month-old WT, OE-1, OE-9, QL-8, and QL-13 plants. Bar = 200 μm; **(B)** Transmission electron microscopy (TEM) examination of the cell wall thickness of xylem fiber cells in 3-month-old WT, OE-1, OE-9, QL-8, and QL-13. Bar = 5 μm. **(C)** Statistical analysis of xylem width in the stems of WT, OE-1, OE-9, QL-8, and L-13 lines. **(D)** Statistical analysis of the cell wall thickness of WT, OE-1, OE-9, QL-8, and QL-13 lines. Student's *t*-test; *P < 0.05; **P < 0.01.

Mutation of particular residues of small GTPases can generate constitutively active forms, which can be used to explore Rab functions. Overexpression of constitutively active RabG3b stimulated autophagy and increased xylem development in *Arabidopsis* and *Populus* (Kwon et al., 2010, 2011). To elucidate the function of *PagRabE1b* in xylem development, transgenic poplars with *PagRabE1b* and *PagRabE1b* (Q74L) overexpression were generated under the control of the CaMV 35S promoter. Two independently transgenic lines (OE-1, OE-9) with higher expression of *PagRabE1b* and two lines with overexpressing a constitutively active form of *PagRabE1b* (Q74L) (QL-8, QL-13) (Zhang et al., 2018b) were selected for further analysis (Figures 2A,B). All WT and transgenic lines were grown in the greenhouse under the same environmental conditions. Compared with the WT, 2-month-old transgenic plants of *PagRabE1b* and *PagRabE1b* (Q74L) exhibited rapid growth and development (Figure 2A). Quantitative measurement of the growth rate and basal stem diameter showed that *PagRabE1b* transgenic plants had a 20.3–28.9% increase in the growth rate and an 11.8–26.5% increase in stem width compared with wild-type plants (Figures 2C,D). To examine whether the xylem development was altered in *PagRabE1b* transgenic poplars, cross-sections of basal stems from 3-month-old WT and *PagRabE1b* transgenic lines were analyzed. Compared with the WT, xylem width was significantly increased in all *PagRabE1b* overexpression lines (Figure 3A). Quantitative analysis showed that the xylem radial width was increased by 27–50% in *PagRabE1b* transgenic plants compared with WT plants (Figure 3B). All these results suggested that *PagRabE1b* may play critical roles in regulating poplar secondary growth and xylem development.

### *PagRabE1b* Overexpression Affects SCW Deposition

Several studies have suggested that RabGTPase has positive roles in the controlling of SCW development. Therefore, we first examined the SCW structure by lignin autofluorescence using CLSM. The results showed that lignification and SCW deposition were increased in *PagRabE1b* transgenic plants than in WT (Supplementary Figure 1). Then, transverse-sections of the 20th internode of 3-month-old transgenic plants were used to examine the SCW structure using TEM. The results showed that the cell walls of xylem fiber cells were thicker in all *PagRabE1b* overexpression plants than in WT plants (Figure 3C). Statistical analysis showed that the SCW thickness of xylem fibers was increased by 10.2, 23.5, 17.65, and 13.1 in-OE-1, OE-9, QL-8, and QL-13 plants, respectively (Figure 3D).

**Figure 4:**
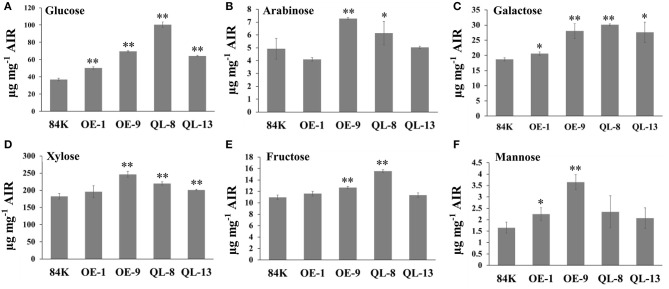
*PagRabE1b* promotes accumulation of monosaccharide content in secondary xylem tissues of transgenic plants. **(A)** Glucose; **(B)** arabinose; **(C)** galactose; **(D)** xylose; **(E)** fructose; **(F)** mannose. Student's *t*-test; *P < 0.05; **P < 0.01.

The differences in SCW thickness may indicate changes in cell wall composition, and therefore, we determined the chemical components of the SCWs using the stem of wild-type and transgenic plants. Overexpression of *PagRabE1b* appeared to have a remarkable effect on monosaccharide contents. The contents of glucose and galactose were significantly increased in all transgenic plants compared with WT. Among these, the content of glucose was increased by 36.1–170% in *PagRabE1b* and *PagRabE1b* (Q74L) overexpression lines than in WT. In addition, the contents of fructose, arabinose, mannose, xylose, and galactose were significantly increased in the *PagRabE1b*or *PagRabE1b* (Q74L) transgenic lines compared with WT (Figure 4). These results suggest that *PagRabE1b* positively regulates secondary wall biosynthesis in *Populus*.

### *PagRabE1b* Promotes the PCD Process in Xylem Formation

**Figure 5:**
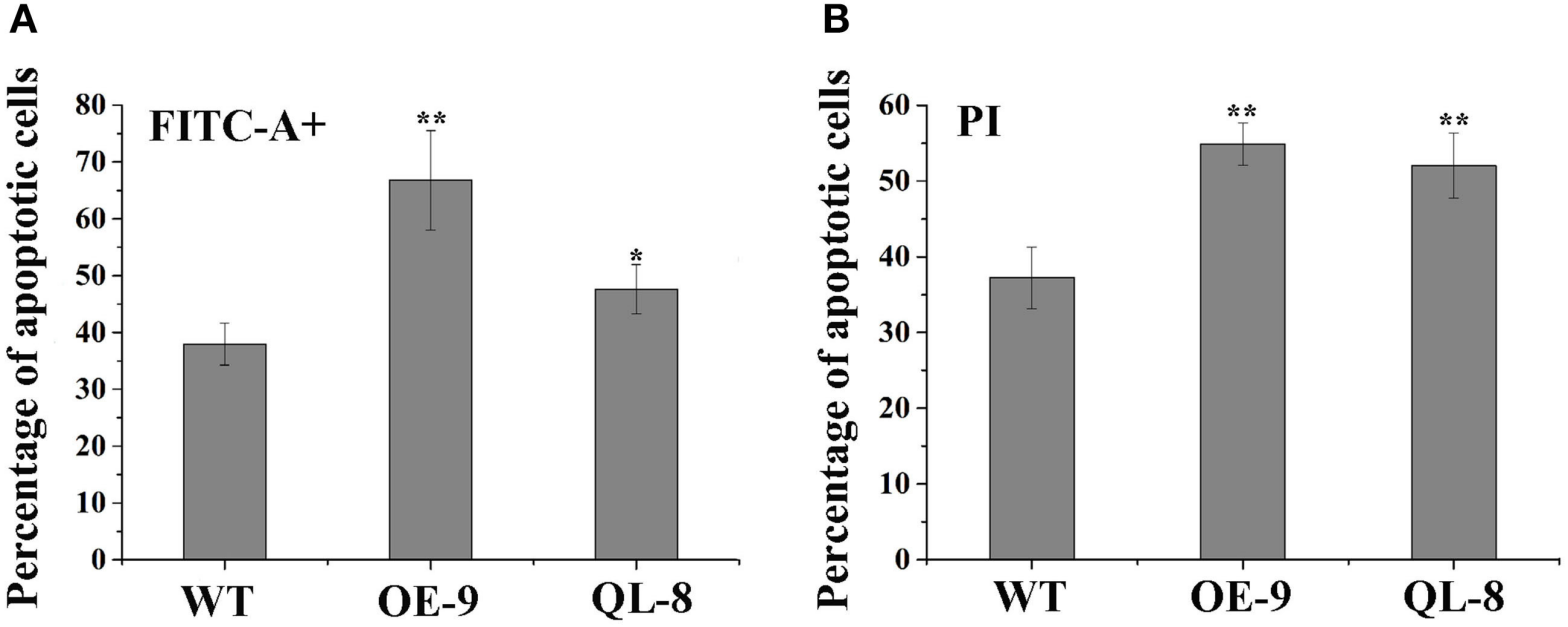
Flow cytometry analyses of the protoplasts from poplar stem-differentiating xylem (SDX) stained with **(A)** annexin V-FITC and **(B)** propidium iodide (PI). Results are means ± SD of three biological replicates. Student's *t*-test; *P < 0.05; **P < 0.01.

Programmed cell death is a pivotal step in xylem formation and is required for the complete maturation of both TEs and fiber cells during xylem development. To investigate the differences of PCD between *PagRabE1b* transgenic and WT plants, protoplasts were isolated from the SDX and analyzed by flow cytometry. Loss of plasma membrane irregularity is one of the earliest characteristics in PCD with exposing phospholipid phosphatidylserine (PS) to the exterior cellular environment. FITC-conjugated Annexin V has a high affinity with PS and acts as a sensitive probe for flow cytometric analysis of the early stage of PCD (O'brien et al., 1998). PI is commonly used to detect dead cells in a population since it is not permeant to live cells. Flow cytometry assay showed that the proportion of apoptotic cells was significantly different in WT and *PagRabE1b* transgenic plants. WT plants contained the lowest percentage (34.3%) of FITC positive cells, while PagRabE1bOE-9 and PagRabE1bQL-8 plants had the higher percentage (56.0 and 45.9%, respectively), of FITC positive cells (Figure 5). Similar to the FITC staining, PagRabE1bOE-9, and PagRabE1bQL-8 plants displayed a significant increase in PI-positive cells (41.5 and 34.8%, respectively), compared with WT plants (20.5%) (Figure 5). This result indicates that *PagRabE1b* has a positive role in PCD during xylem development.

### Expression Analysis of Xylem Development-Related Genes

**Figure 6:**
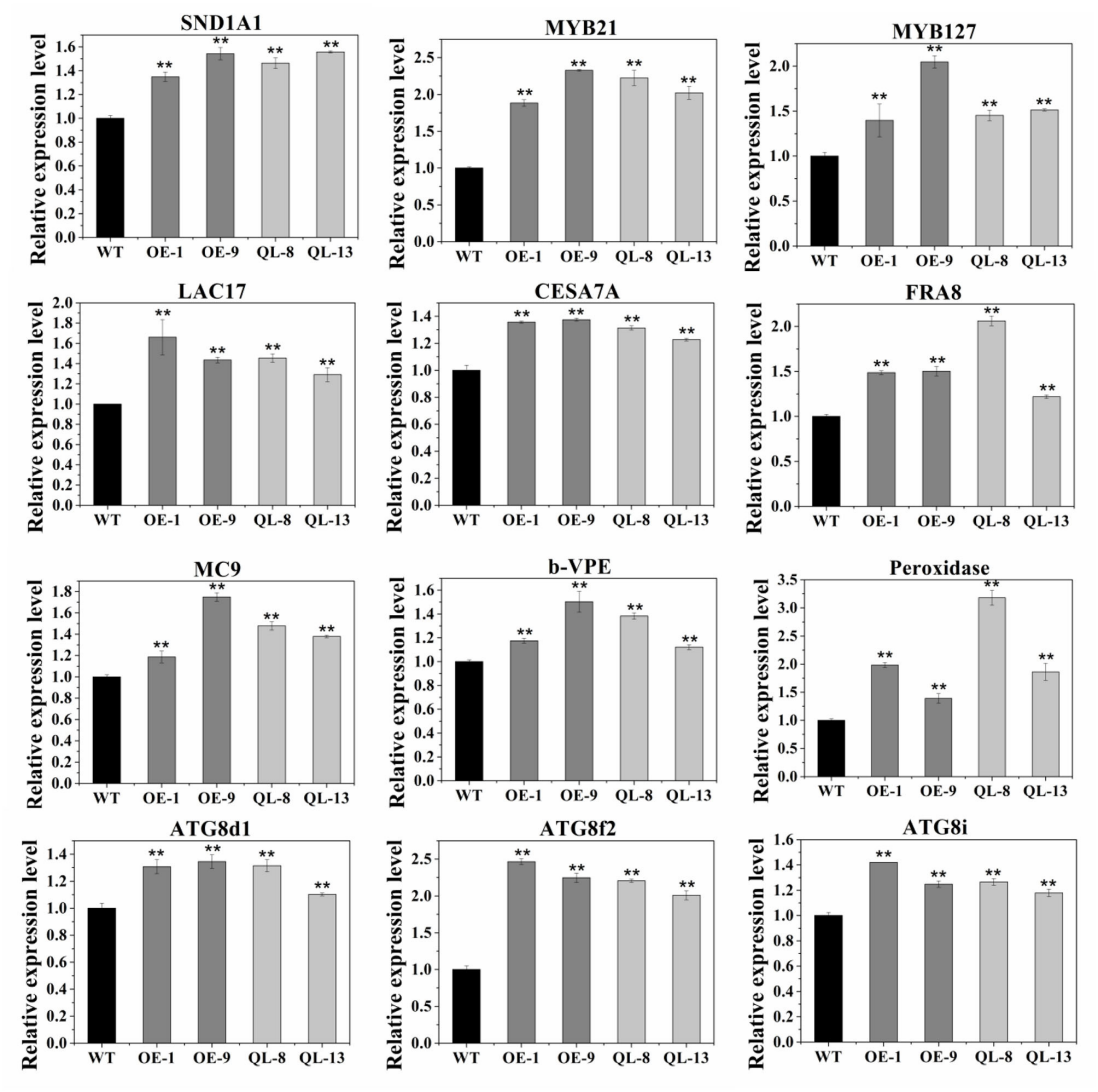
Expression analysis of xylem development-related genes in WT, OE-1, OE-9, QL-8, and QL-13 lines. The poplar actin gene was used as an internal control. Results are means ± SD of three biological replicates. Student's *t*-test; **P < 0.01.

Since manipulation of *PagRabE1b* expression resulted in changes in SCW thickness and PCD of xylem cells, we examined the expression of key genes involved in wood formation by qRT-PCR. As expected, xylem differentiation- and secondary wall regulation-related NAC and MYB genes, such as *SND1-A1* (ortholog of *AtSND1*), *VND6-C1* (*AtVND6*), *MYB21* (*AtMYB46* and *AtMYB83*), *MYB031* (*AtMYB69*), *MYB090* (*AtMYB52* and *AtMYB54*), *MYB127* (*AtMYB67*), and *MYB128* (*AtMYB103*), were upregulated (Figure 6, Supplementary Figure 2). SCW synthesis-related genes containing homologs of *Arabidopsis CESA4, CESA7*, CESA8, *LAC4*, and *LAC17* were also upregulated. Additionally, we also examined the expression levels of PCD-related genes (*Peroxidase, VPE, MC9ATG8d1, ATG8f2*, and *ATG8i*) (Kwon et al., 2011) (Figure 6, Supplementary Figure 2). All these genes were upregulated in OE-1, OE-9, QL-8, and QL-13 plants. These results suggest that *PagRabE1b* overexpression can activate the expression of wood formation-related genes in transgenic plants.

## Discussion

Wood formation is a complicated biological process, including the division and differentiation of the vascular cambium, cell elongation, SCW deposition, and PCD. Although the transcriptional regulatory network was outlined on the SCW synthesis of xylem cells (Zhang et al., 2018a), the precise regulatory mechanisms of xylem cell differentiation during xylem formation remain elusive. Previous research suggests that small GTPases may activate autophagy during wood formation (Kwon et al., 2011), other than regulate SCW development in the xylem of higher plants (Oda and Fukuda, 2014). Therefore, in this study, we further investigated the function of *PagRabE1b* in xylem development in poplar.

Eight of the 11 ROP genes in *Arabidopsis* were expressed in xylem tissues (Winter et al., 2007). In poplar, more than half of the *PtRab* genes were highly expressed in phloem and xylem (Zhang et al., 2018b). In this study, the qRT-PCR analysis showed that *PagRabE1b* was highly expressed in xylem and phloem–cambium (Figure 1). Similarly, the *Eucalyptus* ROP GTPase gene was found preferentially expressed in the cambium area and developing xylem (Foucart et al., 2009). Since cambium includes newly differentiating xylem cells (close to developing xylem) with higher expression than other cell types as shown in Figure 1, we propose that the involvement of *PagRabE1b* gene in the process of xylem development.

To investigate the role of *RabE1b* in woody plants, transgenic poplars overexpressing *PagRabE1b* were generated. The overexpression of *PagRabE1b* leads to an increase in both plant length and stem thickness in poplar (Figure 2). These phenotypic alterations are similar to that with *Arabidopsis RabG3b* overexpression (Kwon et al., 2011). In contrast, the reduced plant size and drastically altered leaf morphology were observed in the *Arabidopsis RabE1d* downregulated transgenic plants (Speth et al., 2009). In addition, virus-induced gene silencing of *NbRabE1* caused various phenotypes, such as growth retardation, premature senescence, and aberrant leaf development (Ahn et al., 2013). These results prove that Rab GTPase plays a pivotal role in plant growth and development.

Phenotypic examination revealed that overexpression of *PagRabE1b* enhanced SCW thickness of fiber cells in poplar stems (Figure 3). Chemical components measurement showed an increase in cell wall constituents in the stems of transgenic plants (Figure 4). To better explain these phenotypes, we used qRT-PCR analyses and found that the expression of marker genes involved in xylem differentiation, cell wall synthesis, and PCD was upregulated in *PagRabE1b* OE plants (Figure 6). The NAC TFs, including SND1 and VND6, were considered as master switches (Zhang et al., 2018b) their orthologs PtrVND6 and PtrSND1 function together for reciprocal cross-regulation of xylem development in poplar (Lin et al., 2017). MYB46 and MYB83 were made up of the second layer of the regulatory network of SCW biosynthesis (Zhang et al., 2018a). Upregulation of which genes increased the biosynthesis of lignin, cellulose, and xylan, and led to the ectopic deposition of SCW (McCarthy et al., 2009; Chai et al., 2014). *LAC17, CESA4A, CESA7A*, and *FRA8* were the enzymes that catalyzed the synthesis of lignin and cellulose. These data indicated that *PagRabE1b* plays a role in xylem differentiation at a higher level, rather than its function in mediating secretory vesicle transportation from the Golgi to the PM to provide substrates for SCW synthesis.

Programmed cell death occurs in the final stage during xylem cell development, which is essential to form empty, water-conducting tracheids or vessels through autolytic processes (Fukuda, 2000). In poplar, autophagosome- and autolysosome-like structures were observed in developing xylem cells in WT, and more accumulation was detected in RabG3bCA overexpression plants (Kwon et al., 2011). In this study, three autophagy-related genes, *ATG8d1, ATG8f2*, and *ATG8i* (Courtois-Moreau et al., 2009), and three PCD-related genes, *peroxidase, VPE*, and *MC9*, were highly expressed in growing stems of *PagRabE1b* OE plants (Figure 6). Flow cytometry analysis showed that the number of apoptotic cells in overexpression transgenic materials was more than that of WT. All these results suggest that *PagRabE1b* positively affects the whole xylem differentiation process, including the final cell lysisin poplar.

In conclusion, the study provided evidence that *PagRabE1b* acts as a positive factor for xylem differentiation and secondary wall formation. This finding provides new information on understanding the regulatory network of wood formation in woody plants.

## Data Availability Statement

The original contributions presented in the study are included in the article/Supplementary Material, further inquiries can be directed to the corresponding author/s.

## Author Contributions

S-TZ conceived and designed the experiments. Y-LL, L-JW, YL, Y-HG, and YC performed the experiments. Y-LL and S-TZ wrote the manuscript. All authors contributed to the article and approved the submitted version.

## Conflict of Interest

The authors declare that the research was conducted in the absence of any commercial or financial relationships that could be construed as a potential conflict of interest.

## Supplementary Material

Supplementary Figure 1 The microscopic images by confocal laser scanning microscopy (CLSM) of Rab transgenic poplars and wild type (WT). **(A,B)** WT; **(C)** OE-1; **(D)** OE-9; **(E)** QL-8; **(F)** QL-13.

Supplementary Figure 2 Quantitative reverse transcription PCR (qRT-PCR) analysis of xylem development-related genes in WT and transgenic lines. The poplar act in the gene was used as an internal control. Results are means ± SD of three biological replicates. Student's *t*-test; ^*^*P* < 0.05; ^**^*P* < 0.01.

Supplementary Table 1 List of primers used in this study.

## References

[B1] Ahn C. S. Han J. A. Pai H. S. (2013). Characterization of in vivo functions of *Nicotiana benthamianaRabE1*. Planta 237, 161–172. 10.1007/s00425-012-1760-5 23001196

[B2] Boerjan W. Ralph J. Baucher M. (2003). Lignin biosynthesis. Annu. Rev. Plant Biol. 54, 519–546. 10.1146/annurev.arplant.54.031902.134938 14503002

[B3] Chai G. Qi G. Cao Y. Wang Z. Yu L. Tang X.. (2014). Poplar *PdC3H17* and *PdC3H18* are direct targets of *PdMYB3* and *PdMYB21*, and positively regulate secondary wall formation in *Arabidopsis* and poplar. New Phytol. 203, 520–534. 10.1111/nph.12825 24786865

[B4] Courtois-Moreau C. L. Pesquet E. Sjodin A. Muniz L. Bollhoner B. Kaneda M.. (2009). A unique program for cell death in xylem fibers of *Populus* stem. Plant J. 58, 260–274. 10.1111/j.1365-313X.2008.03777.x 19175765

[B5] Du J. Gerttula S. Li Z. Zhao S. T. Liu Y. L. Liu Y.. (2020). Brassinosteroid regulation of wood formation in poplar. New Phytol. 225, 1516–1530. 10.1111/nph.15936 31120133

[B6] Foucart C. Jauneau A. Gion J. M. Amelot N. Martinez Y. Panegos P.. (2009). Overexpression of *EgROP1*, a *Eucalyptus* vascular-expressed Rac-like small GTPase, affects secondary xylem formation in *Arabidopsis thaliana*. New Phytol. 183, 1014–1029. 10.1111/j.1469-8137.2009.02910.x 19549133

[B7] Fukuda H. (2000). Programmed cell death of tracheary elements as a paradigm in plants. Plant Mol. Biol. 44, 245–253. 10.1007/978-94-010-0934-8_1 11199386

[B8] Hall A. (1998). Rho GTPases and the actin cytoskeleton. Science 279, 509–514. 10.1126/science.279.5350.509 9438836

[B9] Kwon S. I. Cho H. J. Jung J. H. Yoshimoto K. Shirasu K. Park O. K. (2010). The Rab GTPase RabG3b functions in autophagy and contributes to tracheary element differentiation in *Arabidopsis*. Plant J. 64, 151–164. 10.1111/j.1365-313X.2010.04315.x 20659276

[B10] Kwon S. I. Cho H. J. Lee J. S. Jin H. Shin S. J. Kwon M.. (2011). Overexpression of constitutively active *Arabidopsis* RabG3b promotes xylem development in transgenic poplars. Plant Cell Environ. 34, 2212–2224. 10.1111/j.1365-3040.2011.02416.x 21895694

[B11] Lin Y. C. Li W. Sun Y. H. Kumari S. Wei H. Li Q.. (2013). SND1 transcription factor-directed quantitative functional hierarchical genetic regulatory network in wood formation in *Populus trichocarpa*. Plant Cell 25, 4324–4341. 10.1105/tpc.113.117697 24280390 PMC3875721

[B12] Lin Y. J. Chen H. Li Q. Li W. Wang J. P. Shi R.. (2017). Reciprocal cross-regulation of VND and SND multigene TF families for wood formation in *Populus trichocarpa*. Proc. Natl. Acad. Sci. U.S.A. 114, E9722–E9729. 10.1073/pnas.1714422114 29078399 PMC5692596

[B13] McCarthy R. L. Zhong R. Ye Z. H. (2009). MYB83 is a direct target of SND1 and acts redundantly with MYB46 in the regulation of secondary cell wall biosynthesis in *Arabidopsis*. Plant Cell Physiol. 50, 1950–1964. 10.1093/pcp/pcp139 19808805

[B14] O'brien I. E. W. Baguley B. C. Murray B. G. Morris B. A. M. Ferguson I. B. (1998). Early stages of the apoptotic pathway in plant cells are reversible. Plant J. 13, 803–814. 10.1046/j.1365-313X.1998.00087.x

[B15] Oda Y. Fukuda H. (2012). Initiation of cell wall pattern by a Rho- and microtubule-driven symmetry breaking. Science 337, 1333–1336. 10.1126/science.1222597 22984069

[B16] Oda Y. Fukuda H. (2014). Emerging roles of small GTPases in secondary cell wall development. Front. Plant Sci. 5:428. 10.3389/fpls.2014.00428 25206358 PMC4143617

[B17] Scheller H. V. Ulvskov P. (2010). Hemicelluloses. Annu. Rev. Plant Biol. 61, 263–289. 10.1146/annurev-arplant-042809-112315 20192742

[B18] Speth E. B. Imboden L. Hauck P. He S. Y. (2009). Subcellular localization and functional analysis of the *Arabidopsis* GTPase RabE. Plant Physiol. 149, 1824–1837. 10.1104/pp.108.132092 19233904 PMC2663744

[B19] Sugiyama Y. Nagashima Y. Wakazaki M. Sato M. Toyooka K. Fukuda H.. (2019). A Rho-actin signaling pathway shapes cell wall boundaries in *Arabidopsis* xylem vessels. Nat. Commun. 10:468. 10.1038/s41467-019-08396-7 30692538 PMC6349933

[B20] Sundell D. Street N. R. Kumar M. Mellerowicz E. J. Kucukoglu M. Johnsson C.. (2017). AspWood: high-spatial-resolution transcriptome profiles reveal uncharacterized modularity of wood formation in *Populus tremula*. Plant Cell 29, 1585–1604. 10.1105/tpc.17.00153 28655750 PMC5559752

[B21] Vernoud V. Horton A. C. Yang Z. Nielsen E. (2003). Analysis of the small GTPase gene superfamily of *Arabidopsis*. Plant Physiol. 131, 1191–1208. 10.1104/pp.013052 12644670 PMC166880

[B22] Wang J. P. Matthews M. L. Williams C. M. Shi R. Yang C. Tunlaya-Anukit S.. (2018). Improving wood properties for wood utilization through multi-omics integration in lignin biosynthesis. Nat. Commun. 9:1579. 10.1038/s41467-018-03863-z 29679008 PMC5910405

[B23] Wang Y. Chen Y. Ding L. Zhang J. Wei J. Wang H. (2016). Validation of reference genes for gene expression by quantitative real-time RT-PCR in stem segments spanning srimary to secondary srowth in Populus tomentosa. PLoS ONE 11:e0157370. 10.1371/journal.pone.0157370 27300480 PMC4907450

[B24] Wang Z. Mao Y. Guo Y. Gao J. Liu X. Li S.. (2020). MYB transcription factor161 mediates feedback regulation of secondary wall-associated NAC-Domain1 family genes for wood formation. Plant Physiol. 184, 1389–1406. 10.1104/pp.20.01033 32943464 PMC7608153

[B25] Winter D. Vinegar B. Nahal H. Ammar R. Wilson G. V. Provart N. J. (2007). An “Electronic Fluorescent Pictograph” browser for exploring and analyzing large-scale biological data sets. PLoS ONE 2:e718. 10.1371/journal.pone.0000718 17684564 PMC1934936

[B26] Xie M. Zhang J. Tschaplinski T. J. Tuskan G. A. Chen J. G. Muchero W. (2018). Regulation of lignin biosynthesis and its role in growth-defense tradeoffs. Front. Plant. Sci. 9:1427. 10.3389/fpls.2018.01427 30323825 PMC6172325

[B27] Zhang J. Li Y. Liu B. Wang L. Zhang L. Hu J.. (2018b). Characterization of the *PopulusRab* family genes and the function of *PtRabE1b* in salt tolerance. BMC Plant Biol. 18:124. 10.1186/s12870-018-1342-1 29914373 PMC6006591

[B28] Zhang J. Serra J. A. A. Helariutta Y. (2015). Wood development: growth through knowledge. Nat. Plants 1:15060. 10.1038/nplants.2015.60

[B29] Zhang J. Xie M. Tuskan G. A. Muchero W. Chen J. G. (2018a). Recent advances in the transcriptional regulation of secondary cell wall biosynthesis in the woody plants. Front. Plant Sci. 9:1535. 10.3389/fpls.2018.01535 30405670 PMC6206300

[B30] Zhao Y. Song X. Zhou H. Wei K. Jiang C. Wang J.. (2020). *KNAT2/6b*, a class I KNOX gene, impedes xylem differentiation by regulating NAC domain transcription factors in poplar. New Phytol. 225, 1531–1544. 10.1111/nph.16036 31257603

[B31] Zhong R. Q. Ye Z. H. (2015). Secondary cell walls: biosynthesis, patterned deposition and transcriptional regulation. Plant Cell Physiol. 56, 195–214. 10.1093/pcp/pcu140 25294860

