# Supplementary material for: A Small Guanosine Triphosphate Binding Protein PagRabE1b Promotes Xylem Development in Poplar
Source: Front Plant Sci. 2021 Jun 4;12:686024. doi: 10.3389/fpls.2021.686024 (PMC8213388; doi:10.3389/fpls.2021.686024)
Supplement: Supplementary Figure 1 — The microscopic images by confocal laser scanning microscopy (CLSM) of Rab transgenic poplars and wild type (WT). (A,B) WT; (C) OE-1; (D) OE-9; (E) QL-8; (F) QL-13. [file Data_Sheet_1.PDF]

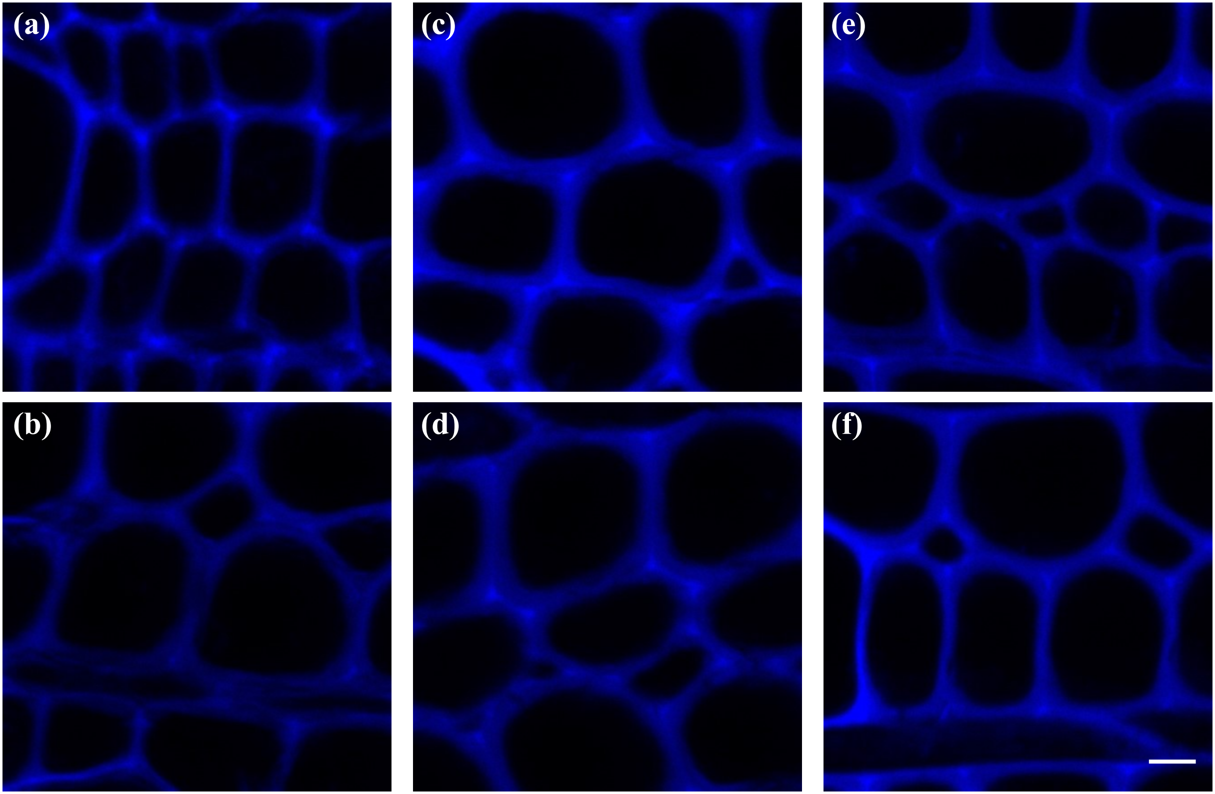

Figure S1.The microscopic images by CLSM of *PagRabE1b* transgenic poplars and WT. in (a-b) Wild-type; (c) *PagRabE1b-OE1;* (d) *PagRabE1b-OE9;* (e) *PagRabE1b-QL8;* (f) *PagRabE1b-QL13*. Bar = 5 μm.
