# Supplementary figures and images for: A Small Guanosine Triphosphate Binding Protein PagRabE1b Promotes Xylem Development in Poplar
Source: Front Plant Sci. 2021 Jun 4;12:686024. doi: 10.3389/fpls.2021.686024 (PMC8213388; doi:10.3389/fpls.2021.686024)

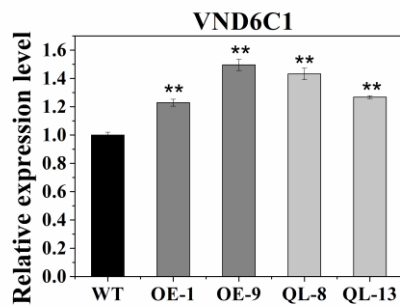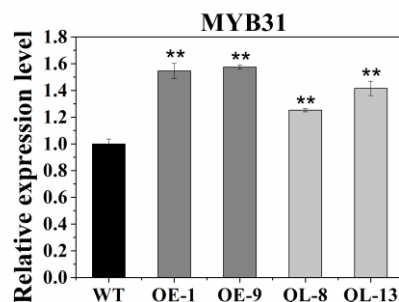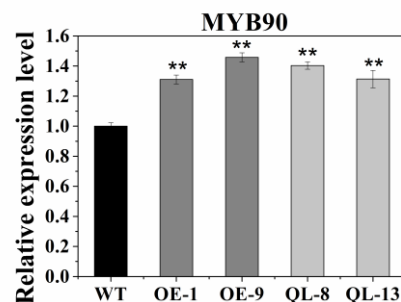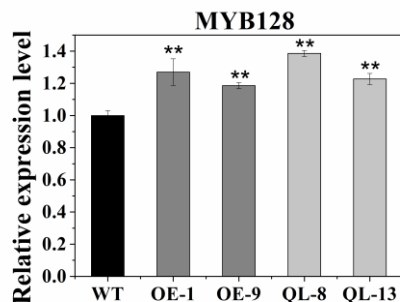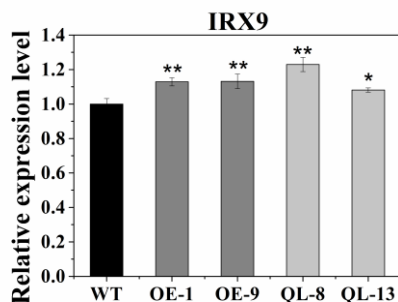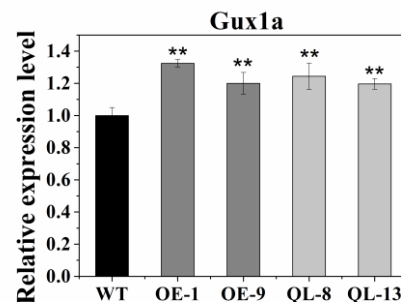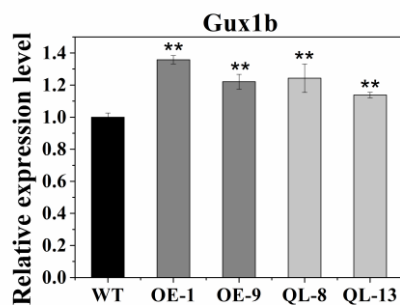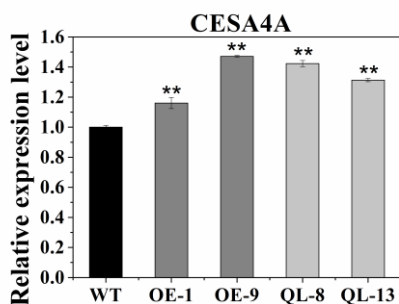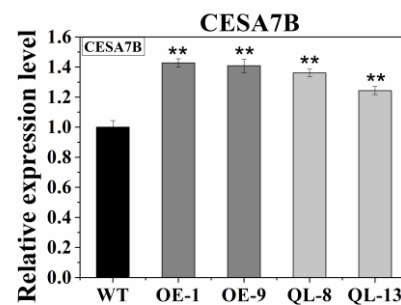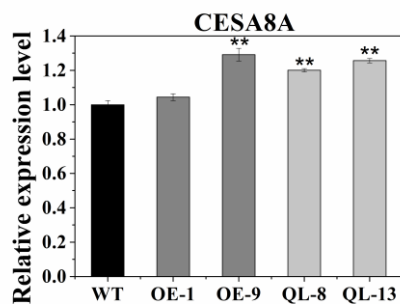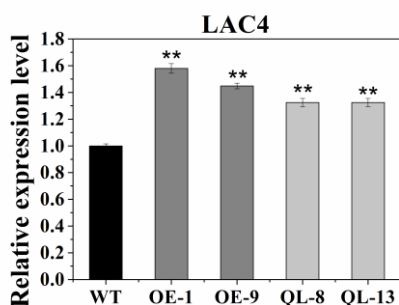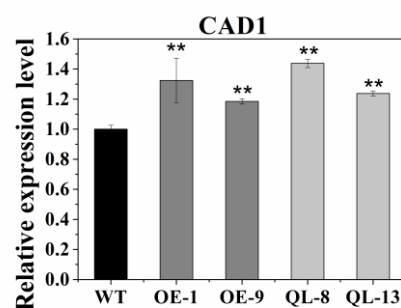

Supplement: Supplementary Figure 2 — Quantitative reverse transcription PCR (qRT-PCR) analysis of xylem development-related genes in WT and transgenic lines. The poplar act in the gene was used as an internal control. Results are means ± SD of three biological replicates. Student's t-test; *P < 0.05; **P < 0.01. [file Data_Sheet_2.PDF]
